# *In Vitro* Cytotoxic Evaluation of MgO Nanoparticles and Their Effect on the Expression of ROS Genes

**DOI:** 10.3390/ijms16047551

**Published:** 2015-04-03

**Authors:** Rangarajulu Senthil Kumaran, Yong-Keun Choi, Vijay Singh, Hak-Jin Song, Kyung-Guen Song, Kwang Jin Kim, Hyung Joo Kim

**Affiliations:** 1 Department of Biological Engineering, Konkuk University, 120 Neungdong-ro, Gwangjin-Gu, Seoul 143701, Korea; E-Mails: dragonrt@konkuk.ac.kr (Y.-K.C.); hjeda11@naver.com (H.-J.S.); hyungkim@konkuk.ac.kr (H.J.K.); 2 Department of Chemical Engineering, Konkuk University, 120 Neungdong-ro, Gwangjin-Gu, Seoul 143701, Korea; E-Mail: vijayjiin@gmail.com; 3 Water Environment Center, Korea Institute of Science and Technology, P.O. Box 131, Cheongryang-Gu, Seoul 130650, Korea; E-Mail: kgsong@kist.re.kr; 4 Urban Agriculture Research Division, National Institute of Horticultural and Herbal Science, Rural Development Administration, Jeonju 560500, Korea; E-Mail: kwangjin@korea.kr

**Keywords:** catalase, nano-genotoxicity, MgO nanoparticle, glutathione *S*-transferase, reactive oxygen species

## Abstract

Water-dispersible MgO nanoparticles were tested to investigate their cytotoxic effects on oxidative stress gene expression. In this *in vitro* study, genes related to reactive oxygen species (ROS), glutathione *S*-transferase (GST) and catalase, were quantified using real-time polymerase chain reactions (molecular level) and molecular beacon technologies (cellular level). The monodispersed MgO nanoparticles, 20 nm in size, were used to treat human cancer cell lines (liver cancer epithelial cells) at different concentrations (25, 75 and 150 µg/mL) and incubation times (24, 48 and 72 h). Both the genetic and cellular cytotoxic screening methods produced consistent results, showing that GST and catalase ROS gene expression was maximized at 150 µg/mL nanoparticle treatment with 48 h incubation. However, the genotoxic effect of MgO nanoparticles was not significant compared with control experiments, which indicates its significant potential applications in nanomedicine as a diagnostic and therapeutic tool.

## 1. Introduction

Advancements in nanotechnology have led to the development of the new field of nanomedicine, which involves the application of nanomaterials and nanodevices for diagnostic and therapeutic purposes. Although nanomaterials are currently being widely used in the fields of life science and biomedicine, there is a serious lack of information concerning the human health and environmental implications of manufactured nanomaterials [1,2]. Recent *in vivo* and *in vitro* studies have suggested that inhalational and dermal absorption of some nanomaterials may have adverse health effects [3] and the use of medical products containing nanomaterials may lead to chronic health conditions [4]. Thus, along with the development of novel nanoparticles, a simultaneous assessment of the toxicological and environmental effects of nanoparticles should be considered [3].

Among various manufactured nanomaterials, metal oxide nanoparticles such as ZnO, Fe_3_O_4_, MnO and MgO have high potential as contrast-enhancement agents for MRI procedures to help provide excellent anatomical images [5,6,7]. A key requirement for the successful use of these nanoparticles in biomedical applications is their good dispersibility, colloidal stability in biological media, internalization efficiency, and low toxicity. Although a variety of methods for synthesizing high-quality metal oxide nanoparticles in organic solvents with improved monodispersity and crystallinity have been reported [8], such nanoparticles are water-immiscible and not sufficiently stable in the physiological conditions. We used the polyehtyleneglycol (PEG)-derivatized phospholipid ligands with biocompatible PEGs as tail groups and surface-coordinating phospholipids as head groups. This sophisticated method produces high water stability and biocompatibility by displacing hydrophobic ligands on the surface of the metal oxide nanoparticles with phospholipids regardless of core materials [9,10].

The potential cytotoxicity of nanoparticles is derived from oxidative stress induced by reactive oxygen species (ROS) [11]. Thus, oxidative stress is a suitable measure for comparing the toxic effects of different nanoparticles [12,13]. In addition, the adverse effects of ROS generation lead to apoptosis, which induces significant cell structure damage to membrane lipids, membrane proteins, and the nuclear membrane [14]. Many studies have demonstrated the ability of nanoparticles to generate ROS in a cell-free environment [15,16]. Some studies have shown that carbon nanoparticles can induce glutathione depletion indicative of oxidative stress in epithelial cell lines [15] and in the lung cells [17]. This oxidative stress has been linked to the induction of the signalling pathways [18] that lead to pro-inflammatory oxidative stress gene expression in macrophages [11]. Therefore, the evaluation of the oxidative potential of nanoparticles is an important part of assessing their toxicity.

In the present study, we selected glutathione *S*-transferase and catalase, which are the most common oxidative stress genes reported in human cell lines [14], to evaluate cytotoxicity through the expression of ROS genes by the engineered metal oxide nanoparticles (MgO), considering the cell viability results of earlier work [7]. Among the metal oxide nanomaterials, MgO is particularly interesting as a low cost, environmentally friendly material. The toxicity of MgO has found to be lower than other metal oxide nanoparticles, including ZnO [19], which is the same size, and TiO_2_ [20], which is commonly attributed to the production of reactive oxygen species [21]. Hence, the PEG-phospholipid encapsulated MgO nanoparticles were used in various concentrations to treat human cancer cell lines with different incubation times. The liver cancer epithelial cells were chosen as *in vitro* models since MRI contrast agents have cytotoxic impact on those cell lines *in vivo*. For quantification of the expressed ROS genes, we analyzed the expression of oxidative stress gene by using both TaqMan probe-based RT-PCR and molecular beacon (MB) technology in the live cells.

## 2. Results

### 2.1. Biocompatible MgO Nanoparticle Synthesis

Monodispersed, water-dispersible MgO nanoparticles were 20 nm in diameter, uniform in size and synthesized by the thermal decomposition of metal-oleate complex. The resulting nanoparticles were stabilized using hydrophobic tails of surfactants (oleic acid and oleylamine) and dispersed in a nonpolar organic solvent such as chloroform. Then, the nanoparticles were encapsulated by PEG-phospholipids shell to endow them with biocompatibility.

### 2.2. Real-Time Quantitation of GST and Catalase Genes

**Figure 1:**
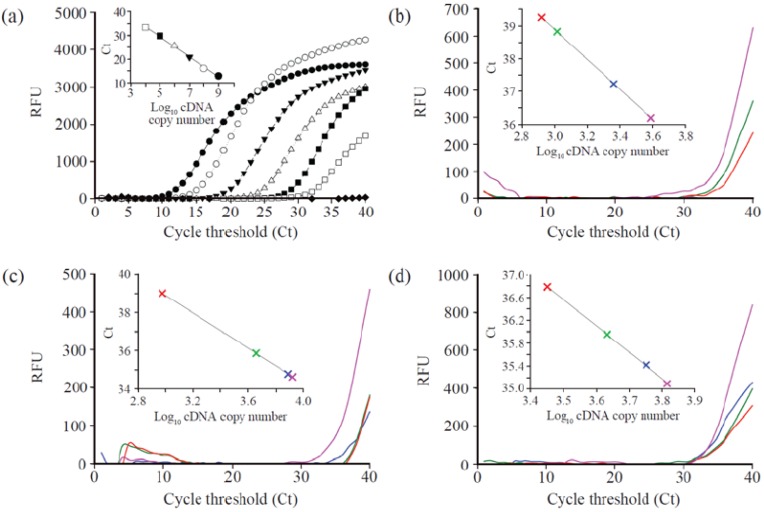
Genotoxicity of MgO nanoparticles on expression of GST in HepG2 cells. (**a**) RT-PCR profiles with serially diluted cDNA as a template (● 1.0 × 10^9^, ○ 1.0 × 10^8^, ▼ 1.0 × 10^7^, △ 1.0 × 10^6^, ■ 1.0 × 10^5^, □ 1.0 × 10^4^, ♦ blank) and the calibration curve of cDNA copy number with respect to the corresponding *C*_t_ values (inset). The enlarged RT-PCR profiles depend on the MgO nanoparticle incubation time of (**b**) 24 h, (**c**) 48 h and (**d**) 72 h, and MgO concentrations (red: control, green: 25 µg/mL, blue: 75 µg/mL, pink: 150 µg/mL).

**Figure 2:**
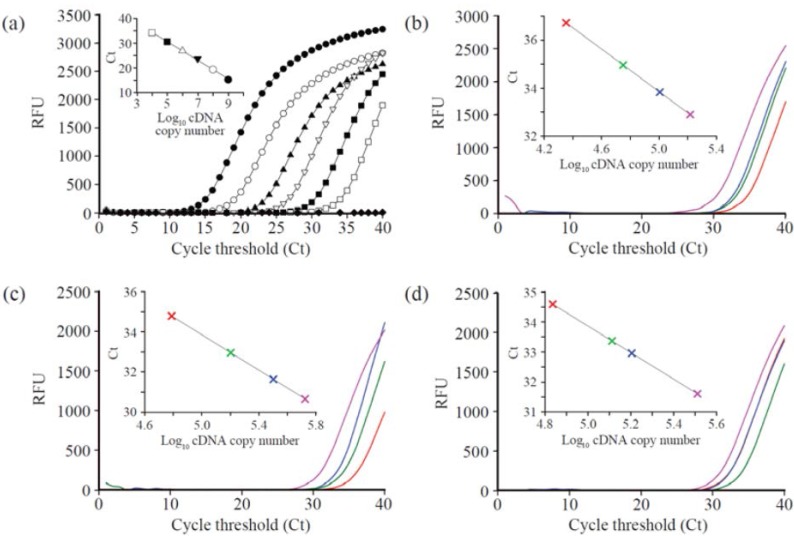
Genotoxicity of MgO nanoparticles on the expression of catalase in HepG2 cells. (**a**) RT-PCR profiles with serially diluted cDNA as a template (● 1.0 × 10^9^, ○ 1.0 × 10^8^, ▼ 1.0 × 10^7^, △ 1.0 × 10^6^, ■ 1.0 × 10^5^, □ 1.0 × 10^4^, ♦ blank) and the calibration curve of the cDNA copy number with respect to the corresponding *C*_t_ values (Inset). The RT-PCR profiles depend on the MgO nanoparticle incubation time of (**b**) 24 h, (**c**) 48 h and (**d**) 72 h, and MgO concentrations (red: control, green: 25 µg/mL, blue: 75 µg/mL, pink: 150 µg/mL).

The genes expressions of the GST and catalase from HepG2 were quantified using RT-PCR. Initially, a calibration curve was obtained by using serial dilution of synthetic cDNA as a template ranging from 10^9^ to 10^4^. Figure 1a and Figure 2a show the calibration curve for GST and catalase gene expression. The RNAs extracted from the cell lines that were treated with 25, 75, and 150 µg/mL of MgO nanoparticles incubated for 24, 48, and 72 h were used for RT-PCR for quantitation. The expressed GST gene level of cells shows the tendency toward decreasing *C*_t_ values as the concentration of nanoparticles increased at a fixed incubation time (Figure 1 and Table 1). At 24 h incubation time, the amount of cDNA gradually increased from 8.40 × 10^2^ for the control to 1.12 × 10^3^ for 25 µg/mL, 2.34 × 10^3^ for 75 µg/mL, and 3.90 × 10^3^ for 150 µg/mL as shown in Figure 1b. At 48 h incubation time (Figure 1c), the corresponding copy amount of cDNA was 9.57 × 10^2^ for a control, 4.60 × 10^3^ for 25 µg/mL, 7.83 × 10^3^ for 75 µg/mL, and 8.40 × 10^3^ for 150 µg/mL. In the case of 25 and 75 µg/mL treatment, GST gene expression decreased slightly, meaning the maximum expression point is located between 24 and 48 h incubation time. However, when 150 µg/mL of MgO was used, the maximum level was near 48 h incubation time due to the relatively high concentration of the MgO treatment. The same or slight decrease in the quantity of cDNA was measured as 4.10 × 10^3^ for 25 µg/mL, 5.37 × 10^3^ for 75 µg/mL, and 5.90 × 10^3^ for 150 µg/mL after 72 h incubation (Figure 1d). Under our experimental conditions, the highest copy number of GST gene in the HepG2 cell was seen to be 8.40 × 10^3^ when treated with 150 µg/mL of MgO at 48 h incubation. A similar trend in gene expression in catalase was observed as shown in Figure 2. This indicates that the expression level increases proportionally with concentration at a fixed incubation time. The maximum gene expression level of the catalase gene was 5.26 × 10^5^ at 150 µg/mL of MgO with 48 h incubation (Figure 2 and Table 2). However, the absolute cDNA copy number with catalase genes were more expressed than GST genes.

**Table 1:** The expressed GST gene number with variation in MgO concentration and incubation time.

MgO Conc. (μg)	cDNA Copy Number		
	24 h	48 h	72 h
0	8.40 × 10^2^	9.57 × 10^2^	2.88 × 10^3^
25	1.15 × 10^3^	4.60 × 10^3^	4.10 × 10^3^
75	2.34 × 10^3^	7.83 × 10^3^	5.37 × 10^3^
150	3.90 × 10^3^	8.40 × 10^3^	5.90 × 10^3^

**Table 2:** The expressed catalase gene number with variation in MgO concentration and incubation time.

MgO Conc. (μg)	cDNA Copy Number		
	24 h	48 h	72 h
0	0.23 × 10^5^	0.62 × 10^5^	0.68 × 10^5^
25	0.57 × 10^5^	1.63 × 10^5^	1.32 × 10^5^
75	1.11 × 10^5^	3.207 × 10^5^	1.61 × 10^5^
150	1.63 × 10^5^	5.26 × 10^5^	3.25 × 10^5^

### 2.3. Quantitative Analysis of GST and Catalase on an Agarose Gel

**Figure 3:**
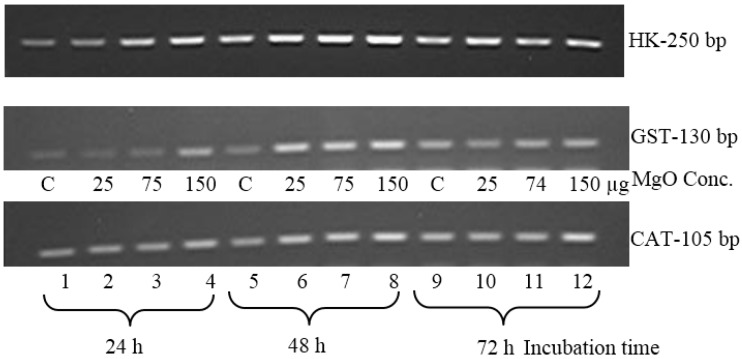
Gene expressions of GST and catalase of HepG2 on agarose gel. Expression of GST and catalase bands of HepG2 cell samples at 30 cycles amplification of cDNA. The gene expressions of GST (130 bp) and catalase (105 bp) showed variations in the band intensity with respect to MgO concentration and incubation time. The house keeping gene (GAPDH) for 200 bp product was also used as a reference control.

The synthesized cDNA of MCF7 cell samples after MgO treatments was amplified by PCR for the expression of GST and catalase with 30 thermal cycles. Figure 3 shows the amplified product bands of GST and catalase genes along the GAPDH housekeeping gene. An increase in the concentration from 25 to 150 µg/mL of MgO nanoparticles showed a progressive increase in the band intensity in the agarose gel. The highest band intensity was expressed in the samples treated with 150 µg/mL of MgO nanoparticles at a fixed incubation time. However, the highest band intensity was observed at 48 h incubation, while the lowest and medium intensity bands were observed at 24 and 72 h, respectively, for both GST and catalase. These results were consistent with the RT-PCR data.

### 2.4. GST Expression in a Living Cell Based on Molecular Beacon Technology

**Figure 4:**
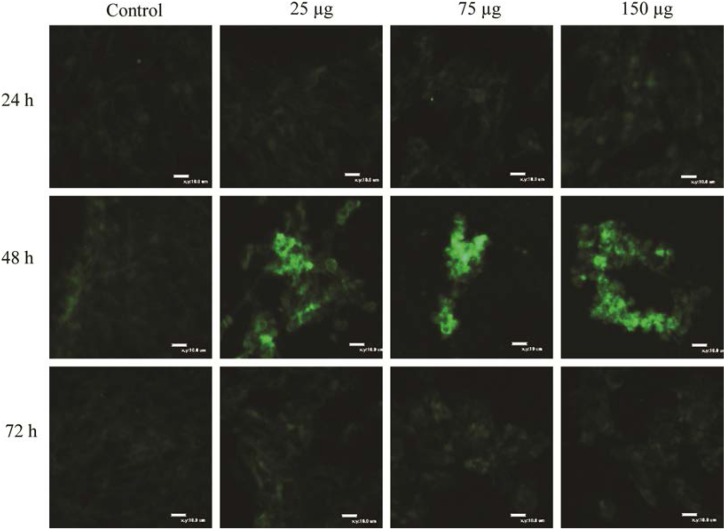
Confocal microscopic images of the expression of GST genes in HepG2 treated with MgO nanoparticles. Molecular beacons targeting the GST gene were delivered into live cells by a reverse permeabilization method using an activated SLO. The fluorescent live cell images at 24 h (**top** panel), 48 h (**middle** panel) and 72 h (**bottom** panel) were presented with excitation at 488 nm and emission detection at 530 nm. The intensity of the fluorescent signals was maximized at 48 h incubation time in proportion to MgO concentrations (scale bar size: 10 µm). The intensity of the fluorescent signals was found to be weak due to the low expression of target genes.

The GST MB probes were delivered into the cytoplasm of cells in a specific growth medium using a reversible permeabilization method with the activation of SLO [22]. Probe hybridization was initiated when the probe was bound with the specific target mRNA for the expression of GST genes. The signals were detected with 488 nm excitation and 530 nm emissions under a laser scanning confocal microscope and a fluorometry. While the fluorescence signals were found to be weak in control cells at all three incubation times, the fluorescence signals were intensified at 24 h incubation, and then decreased at 48 h in T98G cells (Figure 4). Increased concentration of MgO (25, 75 and 150 µg/mL) generated higher fluorescence intensities. The cells displayed green fluorescence signals exterior to the nucleus in the cytoplasm region. This showed that the cells cannot be consistent with the results of quantitative real-time PCR analysis. 

**Figure 5:**
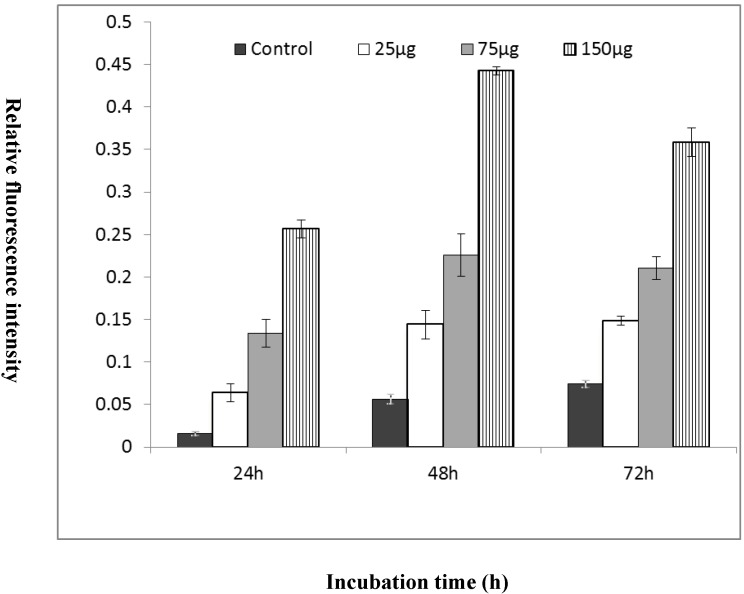
Analysis of gene expression of GST genes of each cell line by fluorometric determination. Fluorometric results showed that the gene expression of the cell line (HepG2), which depends on the MgO concentration and incubation times. Molecular beacons targeting the GST gene were delivered into live cells by a reverse permeabilization method using an activated SLO. The GST gene was expressed maximum at 48 h incubation time with proportion to MgO concentrations.

Fluorometric analysis measures relative fluorescence intensities depending on the incubation time and concentrations of treated MgO nanoparticles (Figure 5). With an increase in MgO concentration from 25 to 150 µg/mL, the fluorescence signals were intensified, and treated cells at 48 h showed higher-intensity expression of fluorescence signals in comparison with 24 and 72 h incubation. At 150 µg/mL MgO concentration, HepG2 cells recorded the strongest fluorescence signal (RFI = 0.44) compared with 24 h (RFI = 0.26) and 72 h (RFI = 0.36) treatments.

## 3. Discussion

Monodispersed MgO nanoparticles synthesized in this study were water-dispersible and biocompatible due to PEG-phospholipid surface modifications. In the case of metal oxide nanoparticles such as iron oxide and manganese oxide, the surface is stable and relatively unreactive compared to other nanoparticles [5,6,7]. Therefore, the strategies are very limited in terms synthesis of water-dispersible metaloxide nanoparticles for biomedical applications [22,23]. Surface modification by PEG-phospholipids utilizes hydrophobic interactions between the surfactants to stabilize the nanoparticles and phospholipids. This does not depend on the core material of nanoparticles, and can therefore be used for any type of inorganic nanoparticle. The high water solubility and biocompatibility of the engineered MgO nanoparticles promotes intracellular uptake and accumulation for imaging cellular distribution and functions. These engineered metal oxide nanoparticles were shown to overcome the drawbacks of currently used MRI contrast agents and can be used as a promising biolabel in molecular and cellular imaging studies [5,7,22]. A nanotoxicological evaluation of the engineered MgO nanoparticles should be performed to ensure biological safety prior to commercialization as MRI contrast agent. Presently, many reports on *in vitro* cytotoxicity studies of nanoparticles are being published, but the wide range of different cell lines, nanoparticle concentrations, exposure times, and colorimetric assays makes it difficult to compare and determine whether the cytotoxicity observed is physiologically relevant. In this study, we screened the cytotoxicity by quantifying the expression of oxidative stress genes (GST and catalase) at the mRNA level using RT-PCR. In this investigation, we have used human cancer cell lines. Cancer cell treatment with nanoparticles may involve unusual physiology in their metabolic process, which induces rapid cell growth and division. This might inhibit normal cell function through increased ROS expression, which ultimately leads to apoptosis.

The quantitative analysis of the GST and catalase genes using RT-PCR has clearly showed that an increase in concentration of MgO was directly proportional to the expression of the oxidative stress genes in all the three cell lines. The cytotoxicity of the cancer cells was maximized at 48 h incubation as compared to 24 and 72 h. One of the possible reasons for this phenomenon is that the accumulation of ROS does not trigger cellular responses at 24 h, but continuous exposure for 48 h increases oxidative stress levels to maximize ROS gene expression. According to the hierarchical oxidative stress hypothesis, at the first tier of oxidative stress, antioxidant response elements are activated, resulting in the expression of phase 2 and antioxidant enzymes such as hemeoxygenase 1 (HO-1), NAD(P)H:quinone oxidoreductase 1 (NQO1), GST, catalase, *etc.* by the transcription factor, Nrf2 [24,25,26]. Then the activity of these enzymes including GST and catalase were augmented to offset the generated ROS in 72 h [26], leading to a decrease in related gene expression levels. Moreover, with a prolonged incubation time of 72 h, cell division was initiated, which reduces the concentration of nanoparticles in the cytoplasm through endocytosis [27]. Thus, a relatively low concentration of nanoparticles has less cytotoxic effect on the newly born-cells, resulting in the reduced quantity of ROS genes at 72 h.

The ROS genes in this study demonstrated that the catalase was found quantitatively with a higher copy number of cDNA than GST. This result suggests that the catalase gene is more regulated by Nrf2 than GST for expressing different levels of antioxidant enzymes under MgO treatment [26,28]. HepG2 cell samples showed the maximum copy number of cDNA in both GST and catalases. This indicates that the quantitation of gene expressions in cell lines play a significant part in assessing the nanotoxic responses in a given time. However, knowledge of the molecular mechanisms that regulate related gene expression depending on cell types is limited. 

The maximum gene expression level (8.40 × 10^3^ for GST and 5.26 × 10^5^ for catalase) of HepG2 cells at 48 h with 150 µg/mL MgO treatment were 9-fold higher in GST and catalase than the control. At 72 h of incubation, the gene expression level (5.90 × 10^3^ for GST and 3.25 × 10^5^ for catalase) was less than twice that of the control. Thus, as the MgO concentration increased to 25, 75, and 150 µg/mL, the gene expression increments were less than two-fold. These results indicate that the PEG-phospholipid coated MgO nanoparticles are stable, biocompatible and less toxic in terms of inducing reactive oxygen species, when considering the nanoparticle concentration and incubation times.

Of all candidate technologies for *in vivo* endogenous mRNA detection, the most promising one is MB technology [29]. We designed the stem and loop sequence to target the GST gene in living cells. To deliver the MB into the cell lines, we used a SLO method, which is rapid, efficient, less damaging and more versatile than the conventional transfection methods. The SLO creates minute pores in the cell membrane through which the MB are delivered into the cells. Once MB targeted its GST mRNA in the cytoplasm, the green fluorescence signal was emitted with 488 nm excitation (Figure 4). In the present experiments, confocal images clearly showed the green fluorescence has a different signal intensity with different concentrations of MgO nanoparticle treatments with incubation time. In the same manner as the RT-PCR, the fluorescence intensities are stronger at 48 h in all the cell lines, and increased with the MgO concentration. As in the established research [16,30] indicating the subcellular localization of metal oxide nanoparticles in BEAS-2B cells induced oxidative stress, it seems that the MgO nanoparticles penetrated the cytoplasm and localized in the peri-regions of the nucleus, which may induce direct interaction between the nanoparticles and cellular molecules to cause adverse toxic cellular responses. We quantified those fluorescence intensities using fluorometric analysis in Figure 5. The GST gene expression trend by MB technology is identical to the RT-PCR data in the three cell lines in terms of the MgO concentration and incubation times, although the intensity ratio based on the control is somewhat different from the values calculated on the basis of cDNA copy number.

## 4. Experimental Section

### 4.1. MgO Nanoparticle Preparation and Cell Culture

Water-dispersible and biocompatible MgO nanoparticles were synthesized (SkySpring Nanomaterials, Inc., Houston, TX, USA) [5,31,32]. Nanoparticles were dispersed in chloroform and then encapsulated by PEG-phospholipids shell to endow them with biocompatibility. Two mL of the organic dispersible oxide nanoparticles in CHCl_3_ (5 mg/mL) were mixed with 1 mL of CHCl_3_ containing 10 mg of 1,2-distearoyl-sn-glycero-3-phosphoethanolamine-*N*-[methoxy(polyethylene glycol)-2000]. After evaporating the solvent, it was incubated at 75 °C in vacuum for 50 min. An addition of 4 mL water resulted in a clear and dark-brown suspension. After filtration and centrifugation, the resulting nanoparticles were then well dispersed in phosphate buffered saline (PBS, pH 7.2). The cell lines were derived from the human hepatocellular carcinoma—HepG2 cells (ATCC HB-8065). The cell lines between 15 and 25 of the subculture passages were used as adherent cells. Cell lines were treated with 25, 75 and 150 µg/mL concentrations of MgO nanoparticles in serum-free culture medium and incubated for a period of 24, 48 and 72 h. The cell lines were grown and maintained in Dulbecco’s Modified Eagle’s Medium (DMEM) was supplemented with 10% fetal bovine serum (FBS) and 1% penicillin (10,000 IU/mL)—streptomycin (10,000 µg/mL). Cells were washed with PBS and incubated in a 3 mL trypsin-ethylenediaminetetraacetic acid (EDTA, 0.2%) for 3–5 min at 37 °C in a CO_2_ incubator. Cell suspensions were then centrifuged for 3 min at 1300 rpm. Then the pellet was re-suspended with a fresh medium and the seed density was adjusted using a hemocytometer based cell counting by an inverted microscope. For the RT-PCR quantitation of ROS, cells were cultured in a 24 micro-well plate (1 × 10^5^ cells/mL).

### 4.2. TaqMan RT-PCR Analysis

The MgO nanoparticle treated cells were used for the isolation of total RNA using a Qiagen RNeasy mini kit. The isolated total RNAs were collected in 20 µL RNase free water and quantified by nanodrop spectrophotometer. We conducted a RT-PCR using a two-step reaction which consisted of complementary DNA (cDNA) synthesis by reverse transcription reaction and then RT-PCR analysis for quantitative gene expression. The cDNA was synthesized by adding 1 µg total RNA to the reverse transcriptase premix tube (AccuPower™ Cyclescript RT premix dT20, Bioneer, Daejeon, Korea) with a reaction volume made up to 20 µL with RNase free water. The tubes were placed in a thermocycler and incubated at 45 °C for 50 min for cDNA synthesis and followed by 90 °C for 5 min for a reverse transcriptase inactivation. Synthesized cDNA was used as a template in the real time analysis. The synthesized primers and TaqMan probes for GST (forward primer: 5'-GATACTGGGGTACTGGGACATCC-3'; reverse primer: 5'-CCACTGGCTTCTGTCATAATCAGG-3', T_m_ 58 °C, product size: 130 bp, gene bank number: NM_146421.2; TagMan probe sequence: 5'-CCCACGCCATCCGCCTGCTCCT-3', T_m_ 68.9 °C ) and catalase (forward primer: 5'-ACAGCAAACCGCACGCTATG-3' with T_m_ 58.6 °C; reverse primer: 5'-CAGTGGTCAGGACATCAGCTTTC-3' with T_m_ 57.3 °C, product size: 150 bp, gene bank number: NM_001752; TagMan probe sequence: 5'-CCCGCTGCTCCTTCCAGTGCTGC-3', T_m_ 69.8 °C ) genes were used in RT-PCR. The TaqMan probes were conjugated with the fluorophore, 5-carboxyfluorescein (FAM) at 5' end and black hole quencher (BHQ1) at 3' end. The 20 µL reaction mixture consisted of 1 µL of cDNA template, 0.25 µL of TaqMan probe (20 µM), 0.5 µL of forward and reverse primers (10 µM), 10 µL of Mastermix (Dynamo™ probe qPCR kit, Finnzymes, Finland) and 7.75 µL of RNase free water. RT-PCR was performed in a qPCR thermocycler (Bio-RAD, Hercules, CA, USA, CFX96TM real-time system) with initiation at 90 °C for 15 min followed by 40 cycles of amplification at 90 °C for 15 s (denature) and 50 °C for 50 s (anneal and elongation). Samples were analyzed with the wavelength of excitation/emission at 488/530 nm with respect to the FAM fluorophore, and the relationship of the amplified cDNA copy number *versus* its cycle threshold (*C*_t_) values was plotted in the graph.

### 4.3. Standard Calibration Curve of RT-PCR

Standard calibration curves for real time analysis were obtained using the total RNA isolated from HepG2 cells. HepG2 cell lines were treated with 1 mM concentration of hydrogen peroxide and incubated for 7 h. Then the isolation of total RNA and reverse transcription reaction were conducted as described above. Special primers for GST (forward primer: 5'-GCACCATGCCCATGATACTG-3' and reverse primer: 5'-ATGCTGCTCCTTCATGCAAC-3') and catalase (forward primer: 5'-GAGGCCTCCTGCAGTGTTCT-3' and reverse primer: 5'-CATTAAGCCATGACGGTGCT-3') were used to obtain a large product (923 bp for GST and 2115 bp for catalase) which is close to the entire gene size (1155 bp for GST and 2300 bp for catalase) in order to mimic real GST and catalase genes. These 923 and 2115 bp product bands were eluted from a gel and quantified using a nanodrop spectrophotometer to determine the exact copy number of the template. Templates were serially diluted from 10^9^ to 10^4^ and used for the RT-PCR reactions to obtain the calibration curve.

### 4.4. Quantitative Analysis on an Agarose Gel

The expression level of GST and catalase genes on an agarose gel was confirmed by PCR. PCR was performed in a 20 µL reaction volume, consisting of 2 µL of templates (cDNA from MCF7 cell line), 1 µL of forward and reverse primer (10 µM), 0.3 µL of enzymes (i-StarMAX™ II DNA polymerase (5 U/mL), Intron Biotech., Sungnam, Korea), 2 µL of dNTP (2.5 mM each), 2 µL of 10× reaction buffer (300 mM Tris-HCl, 300 mM salts consisting of K^+^, NH_4_^+^ and 20 mM Mg^2+^), and 11.7 µL of RNase water. PCR was then carried out in a thermocycler at 90 °C for 2 min, followed by 30 cycles of amplification at 95 °C for 60 s, 55 °C for 30 s and 70 °C for 5 min. The amplified products were then separated in a 1.5% agarose gel electrophoresis with 1× TBE buffer (87 mM Tris base, 90 mM boric acid, and 2 µM EDTA) at 100 V for 60 min. The gel was stained with ethidium bromide (EtBr) and illuminated under UV for detecting the expressed gene bands. A 200 bp housekeeping gene, glyceraldehyde 3-phosphate dehydrogenase (GAPDH) (forward primer: 5'-GCCATCAATGACCCCTTCAT-3' and reverse primer: 5'-GCTCCTGGAAGATGGTGATG-3') were used as a reference control.

### 4.5. Cellular Genotoxicity Test Using MB Technology

The MBs for GST gene expression were designed as 5'-CGCGATC*ACGAAGGATAGTGGGTAGCTGAGGC*GATCGCG-3' with 25 bp loop (italic) and 7 bp stem sequence. A FAM fluorophore and a quencher BHQ1 were labeled at 5' and 3' end, respectively (Bioneer, Korea). Cell lines were cultured in a 96 micro-well plate at a concentration of 5 × 10^4^ cells/mL. After 24 h of incubation, the cells were treated with various concentrations of MgO nanoparticles and different incubation times as described earlier. MBs were delivered into the live cells with a reverse permeabilization method by activated Streptolysin O (SLO) [33]. First the SLO was activated by adding 5 mM of *tris*(2-carboxyethyl)phosphine (TCEP) to 2 U/mL of SLO for 30 min at 37 °C. Then, the cells were incubated for 10 min in 200 µL of serum free medium containing 0.2 U/mL of activated SLO and 3 µL of MB (1 µM) for cell permeabilization and MB delivery. After incubating with MB, the cells were resealed by adding 0.5 mL of the typical growth medium and incubated for 1 h at 37 °C before performing confocal microscopic and fluorometric analysis. The green fluorescence imaging of live cells was performed by a laser scanning confocal microscope (Nikon ECLIPSE C1si, Melville, NY, USA). The culture plates were read under a microplate spectrofluorometer (Spectra Max M2, Molecular Devices, Silicon Valley, CA, USA). The fluorescence signal of the MBs which were hybridized with a GST target gene was analyzed with excitation at 488 nm and emission detection at 530 nm.

## 5. Conclusions

In conclusion, we have evaluated the cytotoxicity of the PEG-phospholipid coated MgO nanoparticles on human cancer cell lines (HepG2) with different concentrations and incubation times. Targeting the two oxidative stress genes (GST and catalase), we performed a quantitative analysis of gene expression at the mRNA level by using the RT-PCR and at the cellular level by using the MB technology. These results are consistent at 48 h incubation, revealing a maximum gene expression proportional to MgO concentration, whereas at 72 h, the gene expression is reduced twice the amount in the control. In addition, the increment ratio of the expressed GST and catalase gene number compared with the control was not significant, demonstrating high stability, biocompatibility, and low toxicity, suggesting that engineered MgO nanoparticles as an excellent contrast agent for magnetic resonance imaging in the field of biomedicine. For further cytotoxicity study, various primary cells and *in vivo* tests also should be performed to explore the linear relationship between gene expression and protein level in cells.
